# SARS-CoV-2 Transmission and Infection Among Attendees of an Overnight Camp — Georgia, June 2020

**DOI:** 10.15585/mmwr.mm6931e1

**Published:** 2020-08-07

**Authors:** Christine M. Szablewski, Karen T. Chang, Marie M. Brown, Victoria T. Chu, Anna R. Yousaf, Ndubuisi Anyalechi, Peter A. Aryee, Hannah L. Kirking, Maranda Lumsden, Erin Mayweather, Clinton J. McDaniel, Robert Montierth, Asfia Mohammed, Noah G. Schwartz, Jaina A. Shah, Jacqueline E. Tate, Emilio Dirlikov, Cherie Drenzek, Tatiana M. Lanzieri, Rebekah J. Stewart

**Affiliations:** ^1^Georgia Department of Public Health; ^2^CDC COVID-19 Response Team; ^3^Epidemic Intelligence Service, CDC.

Limited data are available about transmission of SARS-CoV-2, the virus that causes coronavirus disease 2019 (COVID-19), among youths. During June 17–20, an overnight camp in Georgia (camp A) held orientation for 138 trainees and 120 staff members; staff members remained for the first camp session, scheduled during June 21–27, and were joined by 363 campers and three senior staff members on June 21. Camp A adhered to the measures in Georgia’s Executive Order* that allowed overnight camps to operate beginning on May 31, including requiring all trainees, staff members, and campers to provide documentation of a negative viral SARS-CoV-2 test ≤12 days before arriving. Camp A adopted most^†^ components of CDC’s Suggestions for Youth and Summer Camps^§^ to minimize the risk for SARS-CoV-2 introduction and transmission. Measures not implemented were cloth masks for campers and opening windows and doors for increased ventilation in buildings. Cloth masks were required for staff members. Camp attendees were cohorted by cabin and engaged in a variety of indoor and outdoor activities, including daily vigorous singing and cheering. On June 23, a teenage staff member left camp A after developing chills the previous evening. The staff member was tested and reported a positive test result for SARS-CoV-2 the following day (June 24). Camp A officials began sending campers home on June 24 and closed the camp on June 27. On June 25, the Georgia Department of Public Health (DPH) was notified and initiated an investigation. DPH recommended that all attendees be tested and self-quarantine, and isolate if they had a positive test result.

A line list of all attendees was obtained and matched to laboratory results from the State Electronic Notifiable Disease Surveillance System^¶^ and data from DPH case investigations. A COVID-19 case associated with the camp A outbreak was defined as a positive viral SARS-CoV-2 test** in a camp A attendee from a specimen collected or reported to DPH from the first day at camp A (June 17 for staff members and trainees; June 21 for campers) through 14 days after leaving camp A (trainees left on June 21; staff members and campers left during June 24–June 27). Out-of-state attendees (27) were excluded from this preliminary analysis. Attack rates were calculated by dividing the number of persons with positive test results by the total number of Georgia attendees, including those who did not have testing results, because negative test results are not consistently reported in Georgia.

A total of 597 Georgia residents attended camp A. Median camper age was 12 years (range = 6–19 years), and 53% (182 of 346) were female. The median age of staff members and trainees was 17 years (range = 14–59 years), and 59% (148 of 251) were female. Test results were available for 344 (58%) attendees; among these, 260 (76%) were positive. The overall attack rate was 44% (260 of 597), 51% among those aged 6–10 years, 44% among those aged 11–17 years, and 33% among those aged 18–21 years (Table). Attack rates increased with increasing length of time spent at the camp, with staff members having the highest attack rate (56%). During June 21–27, occupancy of the 31 cabins averaged 15 persons per cabin (range = 1–26); median cabin attack rate was 50% (range = 22%–70%) among 28 cabins that had one or more cases. Among 136 cases with available symptom data, 36 (26%) patients reported no symptoms; among 100 (74%) who reported symptoms, those most commonly reported were subjective or documented fever (65%), headache (61%), and sore throat (46%).

**TABLE:** SARS-CoV-2 attack rates*^,^^†^ among attendees of an overnight camp, by selected characteristics ― Georgia, June 2020

Characteristic	No.^§^	No. positive	Attack rate, %
**Total**	**597**	**260**	**44**
**Sex**			
Male	267	123	46
Female	330	137	42
**Age group, yrs**			
6–10	100	51	51
11–17	409	180	44
18–21	81	27	33
22–59	7	2	29
**Type of attendee (dates attended camp)**			
Trainee (June 17–21)	134	26	19
Staff member (June 17–27^¶,^**)	117	66	56
Camper (June 21–27^¶^)	346	168	49
**Cabin size during camp^††^ (no. of persons/cabin)^§§^**			
Small (1–3)	13	5	38
Medium (7–13)	75	29	39
Large (16–26)	375	200	53

**Abbreviation:** COVID-19 = coronavirus disease 2019.

* Although positive and negative test results for Georgia residents are reportable in the state of Georgia, negative results are not consistently reported. Attack rates were calculated by dividing the number of persons with a positive test result reported to the Georgia Department of Public Health (DPH) by the total number of Georgia attendees, including those who did not provide testing results.

^†^ A COVID-19 case associated with the camp outbreak was defined as a positive viral SARS-CoV-2 test in an attendee from a specimen collected or reported to DPH from the first day at camp A (June 17 for staff members, including trainees; June 21 for campers) through 14 days after leaving camp A (trainees left on June 21; staff members and campers left during June 24–June 27).

^§^ Out-of-state attendees’ (n = 27; 4%) test results were not reported to DPH and therefore were not included in this analysis.

^¶^ Camp departures began June 24 and were completed June 27.

** Three staff members arrived June 21.

^††^ Among camp attendees during June 21–27 (n = 463).

^§§^ No cabins included 4–6 or 14–15 persons.

The findings in this report are subject to at least three limitations. First, attack rates presented are likely an underestimate because cases might have been missed among persons not tested or whose test results were not reported. Second, given the increasing incidence of COVID-19 in Georgia in June and July, some cases might have resulted from transmission occurring before or after camp attendance.^††^ Finally, it was not possible to assess individual adherence to COVID-19 prevention measures at camp A, including physical distancing between, and within, cabin cohorts and use of cloth masks, which were not required for campers. 

These findings demonstrate that SARS-CoV-2 spread efficiently in a youth-centric overnight setting, resulting in high attack rates among persons in all age groups, despite efforts by camp officials to implement most recommended strategies to prevent transmission. Asymptomatic infection was common and potentially contributed to undetected transmission, as has been previously reported (*1*–*4*). This investigation adds to the body of evidence demonstrating that children of all ages are susceptible to SARS-CoV-2 infection (*1*–*3*) and, contrary to early reports (*5*,*6*), might play an important role in transmission (*7*,*8*). The multiple measures adopted by the camp were not sufficient to prevent an outbreak in the context of substantial community transmission. Relatively large cohorts sleeping in the same cabin and engaging in regular singing and cheering likely contributed to transmission (*9*). Use of cloth masks, which has been shown to reduce the risk for infection (*10*), was not universal. An ongoing investigation will further characterize specific exposures associated with infection, illness course, and any secondary transmission to household members. Physical distancing and consistent and correct use of cloth masks should be emphasized as important strategies for mitigating transmission in congregate settings.

## Footnotes

* https://gov.georgia.gov/document/2020-executive-order/06112001/download.

† Notable adopted measures included cohorting of attendees by cabin (≤26 persons), staggering of cohorts for use of communal spaces, physical distancing outside of cabin cohorts, and enhanced cleaning and disinfection, especially of shared equipment and spaces.

§
https://www.cdc.gov/coronavirus/2019-ncov/community/schools-childcare/summer-camps.html

¶ http://sendss.state.ga.us/.

** CDC defines a viral test as one that detects SARS-CoV-2 nucleic acids (e.g., polymerase chain reaction) or antigens. https://www.cdc.gov/coronavirus/2019-ncov/hcp/testing-overview.html.

†† https://dph.georgia.gov/covid-19-daily-status-report.
